# Centrifugal gravity-induced BMP4 induces chondrogenic differentiation of adipose-derived stem cells via SOX9 upregulation

**DOI:** 10.1186/s13287-016-0445-6

**Published:** 2016-12-08

**Authors:** Yeonsue Jang, Hyerin Jung, Yoojun Nam, Yeri Alice Rim, Juryun Kim, Sang Hoon Jeong, Ji Hyeon Ju

**Affiliations:** 1 CiSTEM Laboratory, Convergent Research Consortium for Immunologic Disease, Seoul St. Mary’s Hospital, College of Medicine, The Catholic University of Korea, Seoul, 137-701 South Korea; 2 Division of Rheumatology, Department of Internal Medicine, Seoul St. Mary’s Hospital, College of Medicine, The Catholic University of Korea, Seoul, 137-701 South Korea

**Keywords:** ACAN, ASCs, BMP4, Centrifugal gravity, Chondrogenic differentiation, COL2A1, Proteoglycan, SOX9

## Abstract

**Background:**

Cartilage does not have the capability to regenerate itself. Therefore, stem cell transplantation is a promising therapeutic approach for impaired cartilage. For stem cell transplantation, in vitro enrichment is required; however, stem cells not only become senescent but also lose their differentiation potency during this process. In addition, cytokines are normally used for chondrogenic differentiation induction of stem cells, which is highly expensive and needs an additional step to culture. In this study, we introduced a novel method to induce chondrogenic differentiation of adipose-derived stem cells (ASCs), which are more readily available than bone marrow-derived mesenchymal stem cells(bMSCs), using centrifugal gravity (CG).

**Methods:**

ASCs were stimulated by loading different degrees of CG (0, 300, 600, 1200, 2400, and 3600 g) to induce chondrogenic differentiation. The expression of chondrogenic differentiation-related genes was examined by RT-PCR, real-time PCR, and western blot analyses. The chondrogenic differentiation of ASCs stimulated with CG was evaluated by comparing the expression of positive markers [aggrecan (ACAN) and collagen type II alpha 1 (COL2A1)] and negative markers (COL1 and COL10) with that in ASCs stimulated with transforming growth factor (TGF)-β1 using micromass culture, immunofluorescence, and staining (Alcian Blue and Safranin O).

**Results:**

Expression of SOX9 and SOX5 was upregulated by CG (2400 g for 30 min). Increased expression of ACAN and COL2A1 (positive markers) was detected in monolayer-cultured ASCs after CG stimulation, whereas that of COL10 (a negative marker) was not. Expression of bone morphogenetic protein (BMP) 4, an upstream stimulator of SOX9, was upregulated by CG, which was inhibited by Dorsomorphin (an inhibitor of BMP4). Increased expression of proteoglycan, a major component of cartilage, was confirmed in the micromass culture of ASCs stimulated with CG by Alcian Blue and Safranin O staining.

**Conclusions:**

Chondrogenic differentiation of ASCs can be induced by optimized CG (2400 g for 30 min). Expression of SOX9 is upregulated by CG via increased expression of BMP4. CG has a similar ability to induce SOX9 expression as TGF-β1.

**Electronic supplementary material:**

The online version of this article (doi:10.1186/s13287-016-0445-6) contains supplementary material, which is available to authorized users.

## Background

Transplantation of chondrogenically differentiated stem cells may be an advanced treatment for damaged articular cartilage [1, 2]. Chondrogenic differentiation of stem cells is known to be induced by mechanical stresses [1, 3]. Centrifugal gravity (CG) is one of the easily usable and controllable mechanical stresses and is generated by centrifugation. However, little is known about whether CG can induce chondrogenic differentiation of stem cells.

Impaired articular cartilage cannot be autonomously (naturally) cured. Human adult articular cartilage has a very limited ability to regenerate due to the lack of a precursor cell supply [4]. To provide precursor cells to articular cartilage via the bloodstream, microfracture surgery has been used; however, it often causes fibrocartilage leading to cartilage degeneration [5]. As an alternative, autologous chondrocyte implantation (ACI) showed a satisfactory clinical outcome [6–8]. However, ACI has some disadvantages such as transplanted cartilage separation, limited sources for articular cartilage isolation, and cartilage degeneration [6, 9, 10]. Most recently, stem cells have been transplanted to regenerate damaged articular cartilage [11]. Under the optimized in vitro culture condition, stem cells can be differentiated into chondrocytes, a major component of cartilage [12, 13]. In addition, bone marrow-derived mesenchymal or adipose-derived stem cells (bMSCs and ASCs) are easily isolated from bone marrow and fat, respectively [14–16]. Despite the aforementioned advantages, the method of stem cell transplantation needs to be improved. As a substitute, bMSCs are now used, but they are hardly sufficiently collected and can unexpectedly differentiate to any lineage other than chondrocyte during in vitro cultivation [17, 18]. For these reasons, we wanted to trigger chondrogenic differentiation of ASCs, relatively abundant stem cells, before transplantation. This may reduce unexpected lineage differentiation of stem cells. Considering these concerns, the period from cell isolation to transplantation should be shortened to increase the success rate of cartilage regeneration.

CG may induce chondrogenic differentiation of stem cells. Mechanical stresses induce chondrogenic phenotypes in various cells. Hydrostatic pressure induces chondrogenic phenotypes in synovium-derived progenitor cells via the MAP kinase/JNK pathway [19]. Mechanical compression induces human bMSC chondrogenesis by upregulating chondrocytic genes [20]. Shear stress contributes to chondrogenesis-related extracellular matrix (ECM) expression in human bMSCs [21]. It was previously reported that CG induces gene expression in cells. In lung epithelial carcinoma cells, expression of interleukin-1β is upregulated by centrifugation [22]. Therefore, as a mechanical stress, CG may induce chondrocytic gene expression in stem cells.

Considering that chondrogenic differentiation of stem cells is induced by mechanical stresses, CG may have a similar ability to induce chondrogenic differentiation of stem cells as transforming growth factor (TGF)-β1, a growth factor normally used for in vitro chondrogenesis of stem cells. Therefore, application of CG may make the stem cells produce chondrogenic transcription factors and ECM proteins. In this study, we demonstrated that CG induced chondrocytic gene expression in human ASCs and compared the effects of CG on chondrogenesis with those of TGF-β1.

## Methods

### Stem cell acquisition and culture

Human ASCs obtained from abdomen fat (n = 3, age 30–54 years) were purchased from Catholic MASTER Cells (Seoul, Korea). All experiments were performed with the ASCs from three different donors (age 30–54 years). The research protocol was granted by the Institutional Review Board of Seoul St. Mary’s Hospital. The cells (P2–3, 1.5–3 × 10^3^/cm^2^) were cultured in growth medium, which consists of low-glucose Dulbecco’s modified Eagle medium (DMEM; Gibco, Grand Island, NY, USA) supplemented with 10% fetal bovine serum (FBS; Gibco) and 1% antibiotic-antimycotic solution (Invitrogen, Grand Island, NY, USA) until the cells reach 80–90% confluency in a humidified 5% CO_2_ incubator at 37 °C.

### CG loading

All experiments were performed with 2–3 passaged ASCs. ASCs (P2–3, 1 × 10^5^ cells/mL) were transferred to a new 15 mL conical tube and centrifuged at various gravitational forces (0, 300, 600, 1200, 2400, and 3600 g) for various durations (0, 5, 15, 30, and 60 min) using a centrifuge (1580MGR, Gyrozen, Seoul, South Korea). After CG loading, the cells were re-plated onto a 60 mm culture dish in monolayer and incubated until the desired time points in growth medium.

### Chondrogenic differentiation

Chondrogenic differentiation was modified from previously reported [23]. For three-dimensional pellet culture, CG-stimulated ASCs were resuspended in defined chondrogenic differentiation medium (CDM; high-glucose DMEM supplemented with 1% FBS, 1% ITS + Premix, 100 nM dexamethasone, 1× MEM Non-Essential Amino Acid solution, 50 μg/mL L-proline, and 1% penicillin/streptomycin). Non-treated cells were resuspended in growth media as a negative control. As a positive control, non-treated cells in a 15 mL conical tube were resuspended in CDM containing 10 ng/mL TGF-β1. The tube was placed in an upright position, and cells were incubated at 37 °C in a humidified incubator containing 5% CO_2_. The medium was changed every 2 or 3 days for 2–3 weeks. L-ascorbic acid 2-phosphate has a short half-life, therefore it was provided at the concentration of 50 ug/ml every media change [24, 25].

### Cell viability assay

Cells stimulated with or without CG were seeded onto a 96-well plate. After 24 h, cell viability was investigated using CCK-8 solution (Dojindo Molecular Technologies, Rockville, MD, USA) according to the manufacturer’s manual. Briefly, CCK-8 solution was added to each well to a final concentration of 10%. After incubation for 4 h, absorbance was measured at 450 nm using an ELISA reader (VersaMax, Molecular Devices, Sunnyvale, CA, USA).

### Histological analysis

The pelleted cells were harvested and then histologically analyzed at 21 days. Micromass pellets were washed twice with 1× phosphate-buffered saline (PBS) and fixed for 24 h in 10% formalin. After fixation, micromass pellets were paraffin-embedded and sectioned (5 μm thick). Micromass pellet sections were deparaffinized, rehydrated, and then washed with PBS. To validate chondrogenic differentiation, sectioned micromasses were stained with 1% Safranin O solution or 3% Alcian Blue for 10 min and counterstained with hematoxylin and eosin solution (Sigma-Aldrich, St. Louis, MO, USA) [23]. The sections were mounted, and images were acquired using an inverted fluorescence microscope (IX-71, Olympus, Center Valley, PA, USA).

### Immunofluorescence assay

Rehydrated micromass pellet sections were deparaffinized, rehydrated, and washed twice in buffer. The slides were incubated in 3% H_2_O_2_ (prepared in 1× PBS) for 15 min and washed with tap water for 15 min. The slides were blocked with blocking buffer (10% normal goat serum prepared in PBS) for 1 h [26]. Anti-collagen type II (COL2A1) (1:100; Abcam, Cambridge, MA, USA) and anti-aggrecan (ACAN) (1:200; GeneTex, Irvine, CA, USA) primary antibodies were applied and incubated according to the manufacturer’s recommended protocol, and the slides were washed four times in buffer. The slides were incubated with a fluorochrome-conjugated secondary antibody diluted in 5% normal goat serum for 1 h at room temperature in the dark and then washed three times in 1× PBS for 5 min each in the dark. The slides were incubated with DAPI (1 μg/mL; Molecular Probes, Waltham, MA, USA) for 10 min, washed twice with 1× PBS for 5 min each, and then visualized using fluorescence microscopy.

### Quantitative and semi-quantitative PCR analysis

Total RNA was extracted from stem cells with or without CG stimulation using TRIzol reagent (Invitrogen, Waltham, MA, USA) according to the manufacturer’s protocol. Total RNA (1 μg) was reverse-transcribed using a RevertAid™ First Strand cDNA Synthesis Kit (Fermentas, Burlington, ON, Canada). For quantitative real-time PCR, cDNA (25 ng) was amplified using SYBR® Green PCR Master Mix (Applied Biosystems, Carlsbad, CA, USA). Primers for GAPDH (P267613), SRY (sex-determining region Y)-box (SOX) 5 (P209528), 5′-AACAAGCACA GATCCCCATTG(dTdT)-3′ (sense) and 5′-ACACCGTAAGTGCTCTGGATA(dTdT)-3′ (antisense), SOX6 (P14 2525), 5′- AAGGCTAAAGGGCCTAAGTGA(dTdT)-3′ (sense) and 5′- TTGATGGCATCTTTGCTCCAG(dTd T)-3′ (antisense) SOX9 (P232240), 5′- CCACCTTCACCTACATGAACC(dTdT)-3′ (sense) and 5′-CTGTGTGT AGACGGGTTGTTC(dTdT)-3′ (antisense) and bone morphogenetic protein (BMP) 4 (P196415) 5′-GACACGG TGGGAAACTTTTGA(dTdT)-3′ (sense) and 5′-GGTAACGATCGGCTAATCCTG(dTdT)-3′ (antisense) were purchased from Bioneer (Daejeon, South Korea). Real-time PCR was performed using a real-time system (Roche, Indianapolis, IN, USA). Mean cycle threshold values from triplicate experiments were used to calculate gene expression normalized to GAPDH as an internal control. For semi-quantitative PCR, 25 ng of cDNA was amplified using Taq polymerase and designed primers. The primer sequences were as follows: COL2A1, 5′-GGCAATAGCAGGTTCACGTACA-3′ (sense) and 5′-CGATAACAGTCTTGCCCCACTT-3′ (antisense); ACAN, 5′-GTGCCTATCAGGACAAGGTCT-3′ (sense) and 5′-GATGCCTTTCACCACGACTTC-3′ (antisense); BMP2, 5′-CCCAGCGTGAAAAGAGAGAC-3′ (sense) and 5′-GAGACCGCAGTCCGTCTAAG-3′ (antisense); COL1A1, 5′-CCCCTGGAAAGAATGGAGATG-3′ (sense) and 5′-TCCAAACCACTGAAACCTC TG-3′ (antisense); COL10A1, 5′-CAGTCATGCCTGAGGGTTTT-3′ (sense) and 5′-GGGTCATAATGCTGTTG CCT-3′ (antisense); and TGF-β1, 5′-TCCTGCTTCTCATGGCCA-3′ (sense) and 5′-CCTCAGCTGCACTTGTA G-3′ (antisense). The intensity of RT-PCR bands was quantified using ImageJ (1.40).

### Western blot analysis

For protein extraction, whole cells were lysed in RIPA buffer (50 mM Tris, pH 7.4, 150 mM NaCl, 5 mM EDTA, 30 mM NaF, 1 mM Na_3_VO_4_, 0.1 mM PMSF, protease inhibitors, and 1% Nonidet-P40). The supernatant was harvested and concentrated using a Vivaspin Turbo 15 (Sartorius Stedim, Gottingen, Germany). For normalization, the same number of cells was plated after CG loading or without CG loading for control, and the GAPDH expression of each condition was investigated using western blot analysis. Protein lysates (30 μg) and concentrated supernatants were resolved by 10–12% SDS-PAGE, transferred to polyvinylidene difluoride membranes (Amersham Pharmacia Biotech, Piscataway, NJ, USA), and then probed with primary antibodies against SOX9 (EMD Millipore, Billerica, MA, USA), BMP4 (Santa Cruz Biotechnology, Inc., Dallas, Texas USA), and GAPDH (Abcam). After extensive washing, primary antibodies were detected by peroxidase-linked IgG (1:5000; Abcam) and visualized using an ECL kit (WESTSAVE GOLD, AbFrontier, Geumcheon-gu, Seoul, South Korea). The intensity of western blot bands was quantified using ImageJ (1.40).

### Statistical analysis

All experiments were separately performed at least three times. Statistical analysis was performed using a one-way analysis of variance (ANOVA) with post hoc Bonferroni correction. The statistical comparison at the same time points between two groups was performed using two-way ANOVA. Data are presented as the mean ± standard deviation. For all tests, *p* < 0.05 was considered to be statistically significant.

## Results

### CG increased SOX9 expression in ASCs

**Fig. 1:**
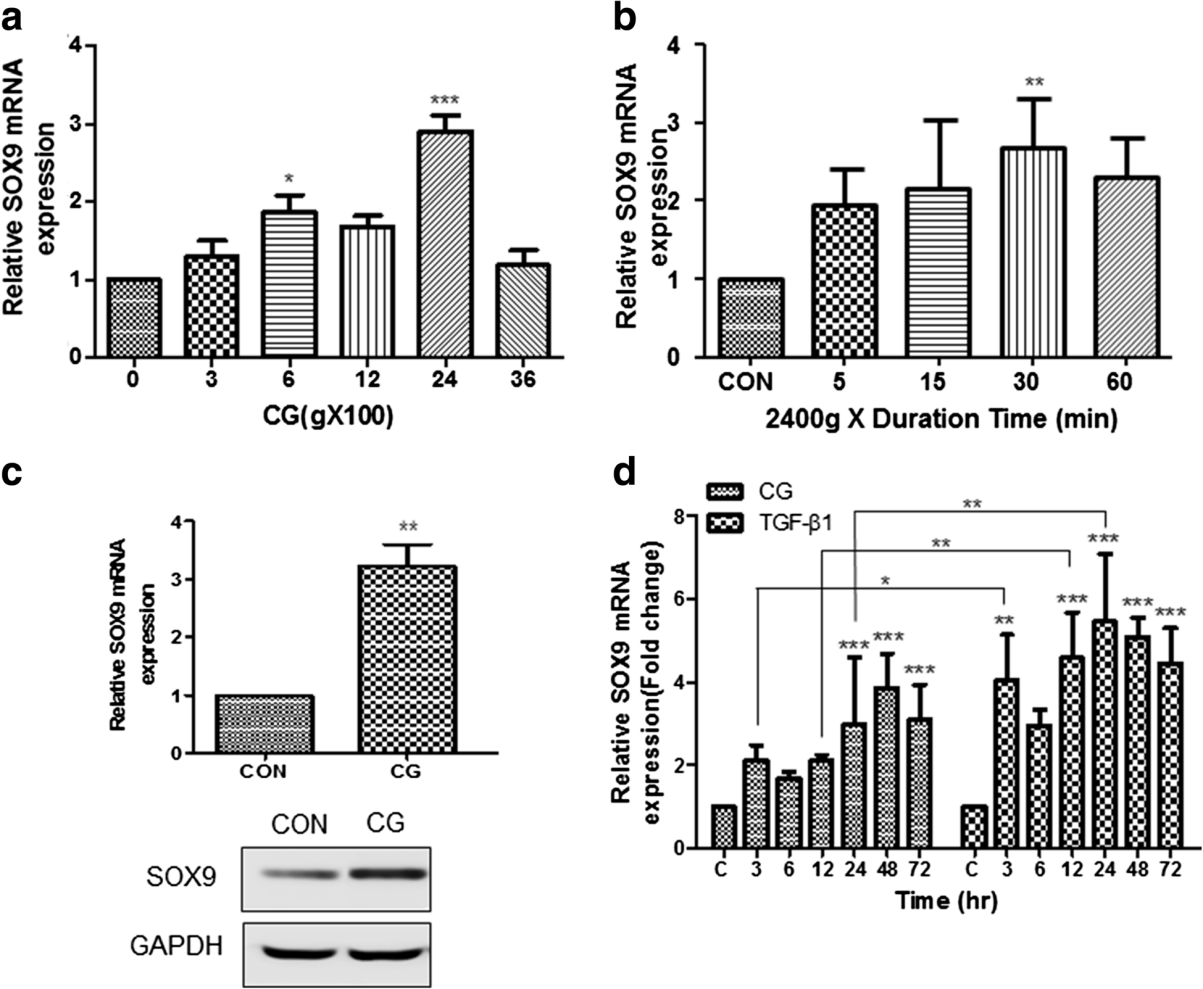
CG increased SOX9 expression in ASCs. Expression of SOX9 mRNA in ASCs stimulated with (**a**) different degrees of CG for 15 min and (**b**) 2400 g for different durations. **c** Expression of SOX9 mRNA and protein in ASCs stimulated with or without 2400 g for 30 min. **d** Comparison of SOX9 mRNA expression in ASCs stimulated with CG or TGF-β1. For CG stimulation, ASCs were centrifuged in a 15 mL tube. ASCs were treated with 10 ng/mL TGF-β1. Expression of SOX9 mRNA and protein was measured by real-time PCR and western blotting, respectively. ^*^ *p* < 0.05, ^**^ *p* < 0.01, and ^***^ *p* < 0.001 for ASCs stimulated with CG versus the control (non-stimulated ASCs) or ASCs treated with 10 ng/mL TGF-β1. *CG* centrifugal gravity, *CON* control, *SOX* SRY (sex-determining region Y)-box, *TGF* transforming growth factor

SOX9 is a master regulator of chondrogenic differentiation [27]. To determine what degree of CG force is suitable to induce SOX9 upregulation, ASCs were stimulated with different degrees of CG (0, 300, 600, 1200, 2400, and 3600 g) for 15 min. After stimulation, the ASCs were re-seeded onto a culture plate and then cultured for 24 h. SOX9 mRNA expression was significantly increased at 600 and 2400 g, and it was approximately threefold higher at 2400 g than in the control (ASCs not loaded with CG) (Fig. 1a). CG-induced SOX9 expression did not show a linear increase. Next, to optimize the CG loading time, SOX9 expression in ASCs stimulated with 2400 g was compared among different time points (0, 5, 15, 30, and 60 min). The expression of SOX9 mRNA was not significantly increased by CG at 5, 15, and 60 min, whereas it was significantly upregulated at 30 min (Fig. 1b). Because CG may affect cell viability, ASC viability was investigated with different loading durations. ASC viability was increased a little after CG stimulation for 5, 15, 30, and 60 min compared to the control, and it was not significantly different between each loading duration time (Additional file 1: Figure S1). Corresponding to SOX9 upregulation by CG stimulation, the expression of SOX9 protein was increased in ASCs centrifuged at 2400 g for 30 min compared with the control (Fig. 1c). TGF-β1 has been used to induce increased expression of SOX9 [28, 29]. Therefore, SOX9 mRNA expression in ASCs stimulated by the optimized CG conditions (2400 g for 30 min) was compared with that in ASCs treated with 10 ng/mL TGF-β1. Upregulation of SOX9 mRNA was observed from 3 h after stimulation with CG or TGF-β1, gradually increased by 24 h, and maintained up to 72 h (Fig. 1d).

### CG-induced SOX9 upregulation was associated with chondrogenic differentiation

**Fig. 2:**
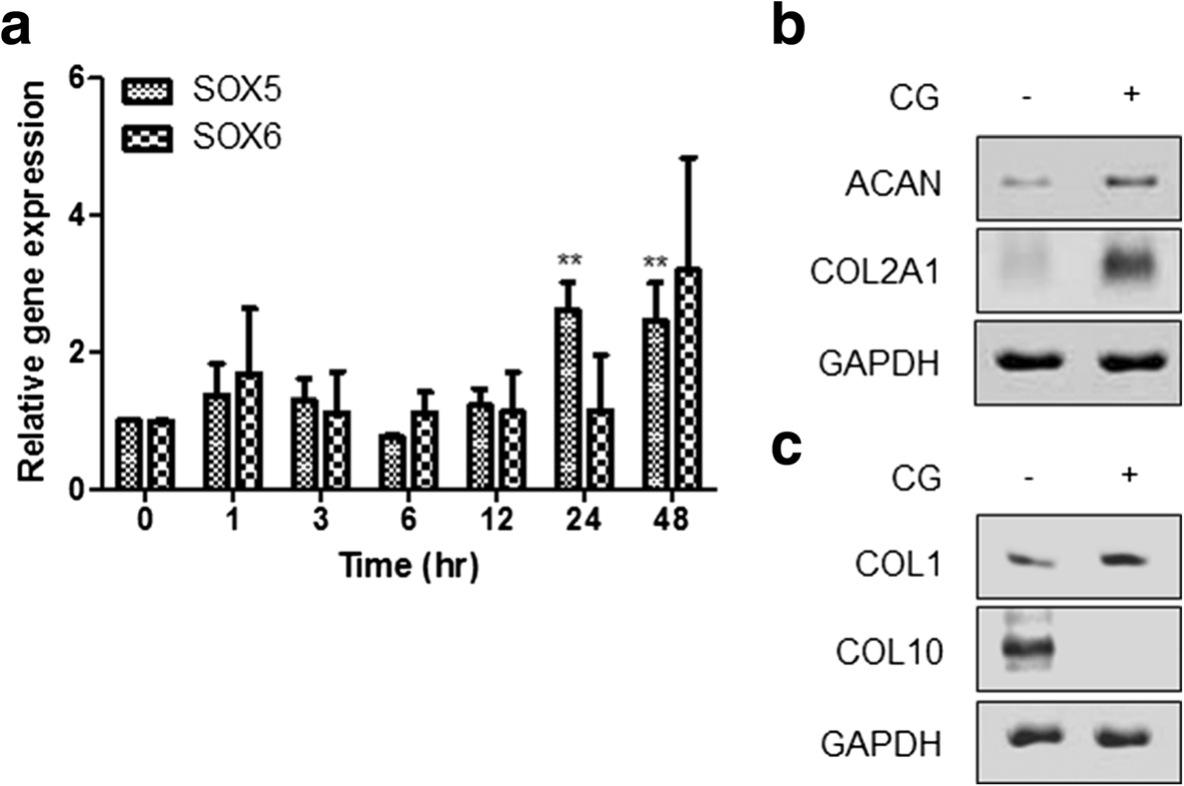
CG-induced SOX9 upregulation was associated with chondrogenic differentiation. **a** Expression of SOX5 and SOX6 mRNA in CG-stimulated ASCs. ASCs were centrifuged at 2400 g for 30 min and then harvested at the indicated time points. ^**^ *p* < 0.01 for ASCs stimulated with CG versus the control (non-stimulated ASCs). Expression of SOX5 and SOX6 mRNA was analyzed using real-time PCR. Expression of (**b**) ACAN and COL2A1 mRNA, and (**c**) COL1 and COL10 mRNA, in ASCs stimulated with or without CG. For CG stimulation, ASCs (P2–3, 1 × 10^5^ cells/mL) were centrifuged in a 15 mL tube at 2400 g for 30 min and then harvested after 24 h. Expression of corresponding genes was measured by reverse transcriptase PCR. *ACAN* aggrecan, *CG* centrifugal gravity, *COL1* collagen type I, *COL10* collagen type X, *COL2A1* collagen type II ​alpha 1, *TGF* transforming growth factor

SOX5 and SOX6 are co-factors involved in chondrogenic differentiation [27, 30]. Expression of SOX5 mRNA was upregulated more than twofold at 24 h after CG stimulation and was maintained up to 48 h (Fig. 2a). On the other hand, expression of SOX6 mRNA was not significantly upregulated by CG stimulation. COL2A1 and ACAN are major components of cartilage as ECM proteins expressed in chondrocytes, and their expression is regulated by SOX9. Expression of ACAN and COL2A1 mRNA was increased by CG stimulation. In particular, COL2A1 expression was greatly increased by CG stimulation (Fig. 2b). In vitro chondrogenic differentiation may be able to induce fibrotic cartilage or hypertrophic differentiation, which inhibits chondrogenic differentiation [31–33]. Therefore, expression of COL1 (a marker of fibrocartilage) and COL10 (a marker of hypertrophy) was examined in ASCs with or without optimized CG stimulation [34–36]. Protein expression of COL1 was slightly increased by CG stimulation, whereas that of COL10 was dramatically decreased (Fig. 2c).

### CG induced SOX9 upregulation via increased expression of BMP4

**Fig. 3:**
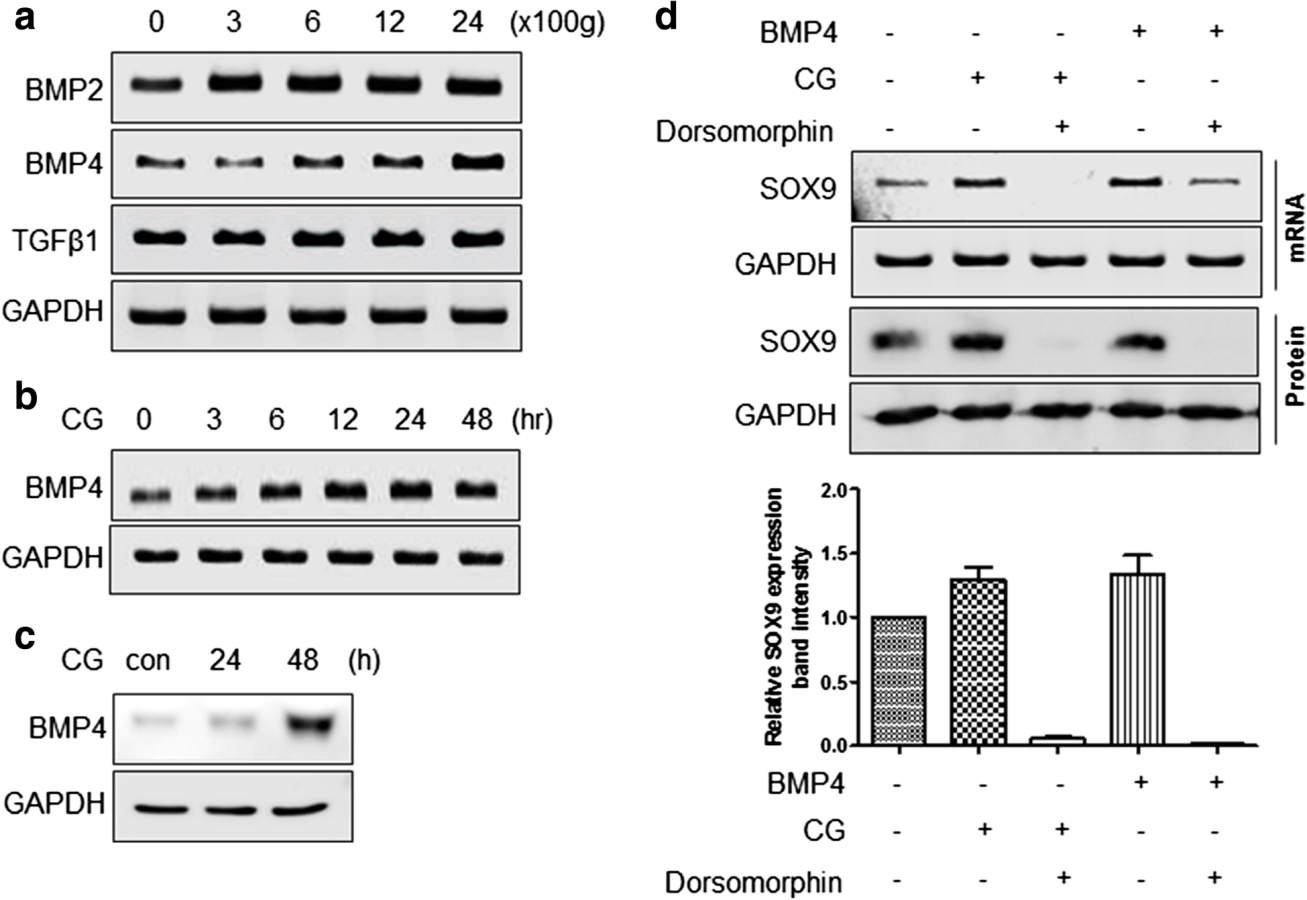
CG induced SOX9 upregulation via increased expression BMP4. **a** Expression of BMP2, BMP4, and TGF-β1 mRNAs in CG-stimulated ASCs. ASCs were loaded with different degrees of CG (0, 300, 600, 1200, and 2400 g) for 15 min. **b** The duration of BMP4 mRNA expression in CG-stimulated ASCs. **c** Expression of BMP4 protein in CG-stimulated ASCs. ASCs were centrifuged at 2400 g for 30 min and then harvested at the indicated time points. Expression of BMP4 mRNA and protein was examined by RT-PCR and western blotting, respectively. **d** BMP4-dependent SOX9 expression in CG-stimulated ASCs. ASCs were stimulated with CG (2400 g for 30 min) and treated with recombinant BMP4 (10 nM) or Dorsomorphin (25 nM). SOX9 protein expression was examined using western blotting. The intensities of the bands in western blots were determined using ImageJ 1.40. *BMP* bone morphogenetic protein, *CG* centrifugal gravity, *SOX* SRY (sex-determining region Y)-box, *TGF* transforming growth factor

BMPs enhance the chondrogenic differentiation induction effect of TGF-β1 [37–39]. In particular, BMP2 and BMP4 are involved in SOX9 expression [40, 41] and are upregulated by a mechanical stress [42]. Expression of BMP2 mRNA was upregulated approximately twofold by CG stimulation, but it differed little among the different CG conditions (Fig. 3a). On the other hand, expression of BMP4 was gradually upregulated by CG (300–2400 g) and showed the greatest increase at 2400 g. Expression of TGF-β1 mRNA was not changed by CG stimulation. Because BMP4 mRNA expression was mainly affected by CG stimulation, its duration was further investigated. Upregulation of BMP4 mRNA was detected from 3 h after CG stimulation, peaked at 24 h, and decreased a little at 48 h (Fig. 3b). Based on the duration of BMP4 mRNA expression, BMP4 protein expression was further examined at 0, 24, and 48 h. Unlike BMP4 mRNA expression, BMP4 protein expression was much higher at 48 h than at 24 h (Fig. 3c). To confirm that increased expression of BMP4 was associated with CG-induced SOX9 upregulation, changes in SOX9 mRNA and protein expression in ASCs were investigated in the presence or absence of CG stimulation, BMP4 recombinant protein, and/or Dorsomorphin (a BMP4 inhibitor). SOX9 upregulation depended on both CG and BMP4. CG-upregulated SOX9 expression was decreased by Dorsomorphin (25 nM) (Fig. 3d).

### CG-stimulated ASCs showed chondrogenic characteristics

**Fig. 4:**
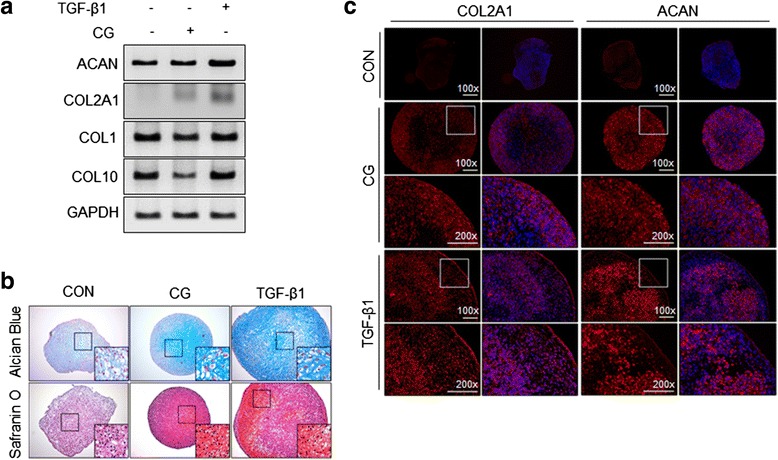
CG-stimulated ASCs showed chondrogenic characteristics. **a** Expression of chondrogenic differentiation markers in ASC micromass cultures. Total RNA was extracted from ASC micromass cultures at 14 days. Expression of each marker mRNA was examined using RT-PCR. **b** Chondrogenic ECM staining of ASC micromass cultures. Sections of ASC micromass cultures at 21 days were stained with Alcian Blue or Safranin O. **c** Expression of ACAN and COL2A1 proteins in ASC micromass cultures. Sections of ASC micromass cultures at 21 days were stained with a fluorescence (phycoerythrin)-conjugated anti-ACAN or COL2A1 antibody (*red*: ACAN or COL2A1, *blue*: DAPI). ASCs were stimulated with CG (2400 g for 30 min) or TGF-β1 (10 ng/mL) and then used for micromass culture. The control was not stimulated with CG or TGF-β1. *ACAN* aggrecan, *CG* centrifugal gravity, *COL1* collagen type I, *COL10* collagen type X, *COL2A1* collagen type II ​alpha 1, *CON* control, *TGF* transforming growth factor

To determine whether CG-stimulated ASCs show a similar level of chondrogenic differentiation as TGF-β1-treated ASCs, marker molecule expression was compared between micromass cultures of ASCs stimulated with CG or TGF-β1. mRNA expression of marker molecules (ACAN and COL2A1 as positive markers; COL1 and COL10 as negative markers) was evaluated after 14 days of chondrogenic differentiation. Compared with the control (non-stimulated micromass culture), ACAN mRNA in micromass cultures was upregulated by TGF-β1, but not by CG. COL2A1 mRNA was upregulated by both CG and TGF-β1, but CG was less effective than TGF-β1. COL1 mRNA was affected by neither CG nor TGF-β1. COL10 mRNA was largely downregulated by CG, but was not affected by TGF-β1 (Fig. 4a). Chondrogenic-specific staining such as Alcian Blue and Safranin O demonstrated that CG stimulation induced a similar level of chondrogenic differentiation of ASCs as TGF-β1. However, the micromass of CG-stimulated ASCs was smaller than that of TGF-β1-treated ASCs (Fig. 4b). To confirm that CG induces chondrogenic phenotypes, the expression of positive markers was further evaluated after 21 days of chondrogenic differentiation using specific antibodies against ACAN and COL2A1. ACAN and COL2A1 proteins were both increased in the micromass of CG-stimulated ASCs, similar to that of TGF-β1-treated ASCs (Fig. 4c).

## Discussion

Centrifugation is a common process in cell harvest and isolation. Therefore, chondrogenic differentiation of stem cells by CG stimulation may be regarded as an unnecessary step because BMP4 and SOX9 upregulation can be induced during cell harvest or isolation. However, expression of SOX9 mRNA was not significantly upregulated at 300 g (Fig. 1a). In general, centrifugation at less than 200 g is used for cell harvest and isolation. It was previously reported that centrifugation at 150–600 g for 5 min can induce chondrogenic differentiation [43–45]. However, such a degree of gravitational force was often used to form a spheroid pellet, which assists the chondrogenic differentiation of stem cells. To induce a similar level of SOX9 expression as that observed upon TGF-β1 treatment, the degree of CG should be around 2400 g and its duration should be 30 min (Fig. 1d). Considering that a centrifuge is a common laboratory device and that 2400 g is easily generated by centrifugation, CG may be an excellent substituent for cytokines, especially TGF-β1, in the chondrogenic differentiation induction of stem cells.

As a rapid response gene, SOX9 expression was significantly increased at 30 min after centrifugation (Fig. 1a). However, for potential clinical application, the duration of CG-induced SOX9 expression was further examined since the longer expression the more advantageous for cartilage generation. As shown in Fig. 1a and b, SOX9 expression at 24 h was approximately three times higher than the control. To objectify the effect of CG on chondrogenic differentiation induction, CG-stimulated ASCs were compared with TGF-β1-treated ones. TGF-β1 is a widely used chondrogenic inducer [46, 47]. In Fig. 1d, SOX9 expression in the ASCs stimulated by CG was generally lower than that by TGF-β1. However, CG-stimulated ASCs showed an average of twofold higher expression of SOX9 than the control ASCs (not stimulated by CG) from 24 h to 72 h, which may mean that CG stimulation is useful to induce the chondrogenic differentiation of ASCs. Indeed, positive markers for chondrogenic differentiation (ACAN and COL2A1) were upregulated by CG in monolayer cultured ASCs, whereas a negative marker (COL10) was downregulated (Fig. 2b and c). The expression of COL10 in the pellet culture of CG-stimulated ASCs was not matched to that of monolayer cultured ones. In the case of monolayer cultured cells, COL10 was not expressed by CG at all: CG downregulated the expression of COL10. On the other hand, in chondrogenic differentiation culture, COL10 was expressed in the cells exposed to CG, but not as high as the control and TGF-β1-treated group. According to previous reports, the expression of COL10 was increased during in vitro chondrogenic differentiation [48]. Considering the lower expression level of COL10 in the in vitro chondrogenic differentiation culture of CG-stimulated ASCs than those of the control and TGF-β1-treated group, CG may have a suppressive effect on COL10 expression. Similar to the traditional culture, chondrogenic-specific matrices were positively stained in the pellet of CG-stimulated ASCs compared to the control (Fig. 4b). Interestingly, the pellet size was markedly larger in the traditional method of pellet culture (namely, TGF-β1-treated ASCs) than that of CG-stimulated ASCs. This may be caused by more stimulation in the traditional method. For the traditional method of culture, medium was changed every other day with fresh TGF-β1, a chondrogenic differentiation inducer for 2–3 weeks. On the other day, fresh medium was provided for the CG group as the traditional method group, but CG stimulation was not (CG was provided only once before pellet culture). As a growth factor, TGF-β1 can increase cell proliferation as well as chondrogenic differentiation. Therefore, the pellet size of the traditional method group was bigger than that of the CG group. In Fig. 4c, the matrix deposition pattern of the CG group was perichondrial distribution, whereas that of the traditional method group was uniform distribution. This difference may be associated with the level of matrix protein expression. The multiple stimulation of TGF-β1 may induce higher expression of matrix protein than CG stimulation, which leads to uniform distribution in the traditional method group. Since the potential application of CG-stimulated ASCs to 1-day surgery was considered, we provided CG stimulation only once, and compared the chondrogenic differentiation ability of CG-stimulated ASCs with that of the traditional method group (continuously stimulated with TGF-β1).

BMPs are associated with chondrogenic differentiation, especially to enhance the function of TGF-β1 in chondrogenic differentiation induction [37, 39]. In the present study, BMP4 was upregulated by CG (Fig. 3a). BMP4 is upregulated by mechanical stress [42] and is also involved in SOX9 expression. Based on our data, CG may be a good substituent for TGF-β1 in that it can be easily generated in a general laboratory environment and requires no expense. For a better understanding of CG-increased BMP4 expression, it should be further studied how CG induces increased expression of BMP4. Furthermore, for clinical applications, the effect of CG-stimulated ASCs on cartilage regeneration needs to be evaluated in an in vivo model.

In our study, we induced chondrogenic differentiation of ASCs using centrifugation, properly speaking, the gravity force generated by centrifugation. Centrifugation is a way to put gravity force on ASCs. Therefore, any type of centrifugation can be used to induce chondrogenic differentiation of ASCs. A more important thing than the type of centrifugation may be the degree and duration time of centrifugal gravity force. Based on our results, centrifugation at 2400 g for 30 min was the most optimal for the induction of SOX9 expression in ASCs. Among the degrees of CG tested, SOX9 expression was induced only at 600 g and 2400 g, which may mean that SOX9 expression is not proportional to the degree of CG. In a previous study, mechanical compression induced the alteration of microRNA expression, which was not proportional to the degree of compression [49]. As shown in Fig. 1a, SOX9 expression was not increased at 3600 g at all. This may be mechanobiological physiology. Interestingly, according to previous studies, mechanical stresses were associated with osteogenic differentiation. Considering that gravity force is one of mechanical stresses, a different degree of centrifugation may induce osteogenic differentiation [50]. In addition, adipogenic and fibrogenic differentiation may be associated with centrifugation. It was reported that mechanical stress suppressed adipogenic differentiation [51], whereas it induced fibrogenic differentiation [52]. Accordingly, CG stimulation may be a useful tool for various lineaged differentiation of stem cells as well as chondrogenic differentiation.

## Conclusions

In this study, we demonstrated that CG can induce chondrogenic differentiation of ASCs via increased expression of BMP4 and SOX9. Our data suggest that CG-stimulated ASCs may improve stem cell-based cartilage regeneration regarding convenience and cost.

## Additional file

Additional file 1: Figure S1. Comparison of the viabilities of ASCs stimulated with CG for different durations. ASCs were stimulated with CG (2400 g) for different durations (0, 5, 15, 30, and 60 min) and then plated onto a 96-well tissue culture plate. At 24 h after CG stimulation, ASCs were incubated with CCK-8 for 4 h and then their viabilities were determined by measuring absorbance at 450 nm using an ELISA reader. All experiments were performed in triplicate. ^***^ *p* < 0.001 for ASCs stimulated with CG versus the control (non-stimulated ASCs). (TIF 1026 kb)

## Abbreviations

ACAN: aggrecan
ACI: autologous chondrocyte implantation
ASC: adipose-derived stem cell
BMP: bone morphogenetic protein
CDM: chondrogenic differentiation medium
CG: centrifugal gravity
COL2A1: collagen type II alpha 1
DMEM: Dulbecco’s modified Eagle medium
ECM: extracellular matrix
FBS: fetal bovine serum
MSC: mesenchymal stem cell
PBS: phosphate-buffered saline
SOX: SRY (sex-determining region Y)-box
TGF: transforming growth factor
