# Supplementary figures and images for: Centrifugal gravity-induced BMP4 induces chondrogenic differentiation of adipose-derived stem cells via SOX9 upregulation
Source: Stem Cell Res Ther. 2016 Dec 8;7:184. doi: 10.1186/s13287-016-0445-6 (PMC5144493; doi:10.1186/s13287-016-0445-6)

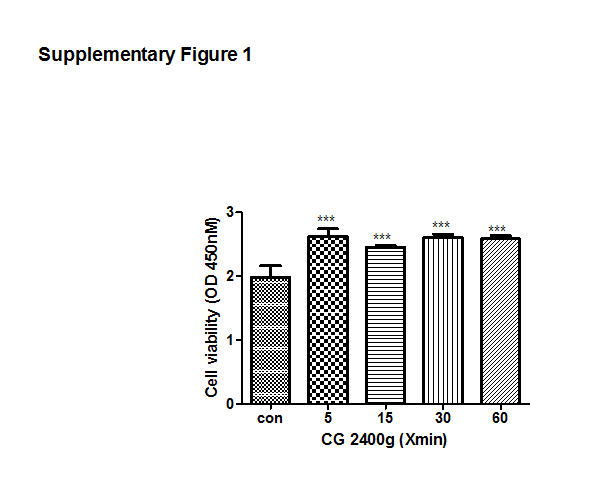

Supplement: Additional file 1: Figure S1. — Comparison of the viabilities of ASCs stimulated with CG for different durations. ASCs were stimulated with CG (2400 g) for different durations (0, 5, 15, 30, and 60 min) and then plated onto a 96-well tissue culture plate. At 24 h after CG stimulation, ASCs were incubated with CCK-8 for 4 h and then their viabilities were determined by measuring absorbance at 450 nm using an ELISA reader. All experiments were performed in triplicate. *** p < 0.001 for ASCs stimulated with CG versus the control (non-stimulated ASCs). (TIF 1026 kb) [file 13287_2016_445_MOESM1_ESM.tif]
